# Inflammation as a diagnostic criterion in the GLIM definition of malnutrition—what CRP-threshold relates to reduced food intake in older patients with acute disease?

**DOI:** 10.1038/s41430-021-00977-4

**Published:** 2021-07-19

**Authors:** Maryam Pourhassan, Tommy Cederholm, Ulrike Trampisch, Dorothee Volkert, Rainer Wirth

**Affiliations:** 1 Department of Geriatric Medicine, Marien Hospital Herne, Ruhr-Universität Bochum, Bochum, Germany; 2 Department of Public Health and Caring Sciences, Clinical Nutrition and Metabolism, Uppsala University, Uppsala, Sweden; 3 Institute for Biomedicine of Aging, Friedrich-Alexander-Universität Erlangen-Nürnberg, Nuremberg, Germany

**Keywords:** Nutrition, Geriatrics

## Abstract

**Background/objectives:**

In the recently introduced GLIM diagnosis of malnutrition (Global Leadership Initiative on Malnutrition), details of how to classify inflammation as an etiologic criterion are lacking. This study aimed to determine at what level of serum C-reactive protein (CRP) the risk of low food intake increases in acutely ill older hospitalized patients.

**Subjects/methods:**

A total of 377 patients, who were consecutively admitted to a geriatric acute care ward, were analyzed. Nutritional intake was determined using the food intake item of Nutritional Risk Screening and the plate diagram method and patients were grouped into three categories as >75%, 50–75% and ≤50% of requirements. CRP was analyzed according to standard procedures and patients were classified into different CRP groups as follows: 0.0–0.99 mg/dl, 1.0–1.99 mg/dl, 2.0–2.99 mg/dl, 3.0–4.99 mg/dl, 5.0–9.99 mg/dl and ≥10.0 mg/dl.

**Results:**

Of the total population (mean age of 82.2 ± 6.6 years; 241 females), 82 (22%) had intake <50% of requirements and 126 (33%) demonstrated moderate to severe inflammation. Patients with food intake <50% of requirements had a significantly higher median CRP level compared to patients with food intake >75% of requirements (*P* < 0.001). The group with serum-CRP levels above 3.0 mg/dl had a markedly higher proportion of patients with low food intake; i.e., <50% and <75% of the requirements.

**Conclusion:**

A serum-CRP of 3.0 mg/dl appears to be a reasonable threshold of acute inflammation leading to reduced food intake to serve as an orientation with regard to the inflammation criterion of the GLIM diagnosis in acutely ill older patients.

## Introduction

Malnutrition with weight and muscle loss is a frequent syndrome in older patients. The etiology of malnutrition in older persons is mostly multifactorial and not entirely understood [1]. In general, malnutrition is caused by an imbalance of energy intake and energy requirements, which in older persons, mostly emerges due to impaired food consumption [2, 3]. Previous studies have demonstrated that disease-related inflammation, besides other causes, frequently diminishes appetite and food intake [4–7], and is therefore an important risk factor of malnutrition. Despite a significant association with adverse functional and clinical outcomes, malnutrition remains underdiagnosed and undertreated in the majority of patients. One reason for the under-recognition of malnutrition may be the previous lack of a uniform definition. Therefore, a new and global diagnostic framework of malnutrition has been proposed by the Global Leadership Initiative on Malnutrition (GLIM) [8]. At least one phenotypic criterion including unintentional weight loss, low body mass index and/or reduced muscle mass, combined with at least one etiologic criterion including reduced food intake, malabsorption or inflammation are required for the diagnosis of malnutrition based on the GLIM criteria [8]. Whereas the criteria of weight loss, body mass index, and low energy-intake are defined with respective thresholds, details of how to classify inflammation as an etiologic criterion are more obscure. Because the etiologic criterion of low food intake is complemented and could be replaced by the criterion of inflammation (if positive) in accordance with the GLIM construct, it is of particular interest to clarify which level of inflammation may markedly increase the risk of low food intake in older patients.

C-reactive protein (CRP) is an unspecific biologic marker of systemic inflammation, which may be elevated in both acute and chronic inflammation [9–11]. Serum-CRP is typically <0.5 mg/dl in healthy persons [12, 13], whereas increased higher serum concentrations have been reported to be, in general, associated with low food intake in hospitalized older patients [4]. Therefore, this pilot study aimed to determine at what level of serum CRP the risk of low food intake increases in acutely ill older hospitalized patients.

## Subjects and methods

This cross-sectional study is a post hoc analysis of two previous studies undertaken at two acute care geriatric hospital departments (Marien-Hospital Herne, MHH and St. Marien-Hospital Borken, MHB) in Germany. Detailed descriptions of the study populations and methods have been reported in more detail elsewhere [4, 5, 14]. Three hundred and seventy-seven patients (200 patients from MHH and 177 patients from MHB) with age range between 63 and 100 years, who were consecutively admitted, were analyzed. In both studies, patients were recruited if they were older than 60 years and able to cooperate. All measurements of this analysis were conducted during the first days after hospital admission. The study protocols were approved by the ethical committee of Ruhr- University Bochum (no. 16-5956, approved on 4 April 2017) and ethical committee of Friedrich-Alexander-University Erlangen-Nürnberg (no. 16_16 Bc, approved on 1 February 2016). Written informed consent was obtained from all patients.

### Assessment of nutritional status and nutritional intake

Nutritional status was evaluated using the Mini Nutritional Assessment Short Form (MNA-SF) [15] and patients were classified as having normal nutritional status (12–14 points), being at risk of malnutrition (8–11 points) and malnourished (0–7 points). Nutritional intake was determined using the food intake item of Nutritional Risk Screening (NRS-2002) [16] in MHB and the plate diagram method [17] in MHH. Using both methods, patients were grouped into three categories, according to nutritional intake >75%, 50–75% and ≤50% of requirements.

### Geriatric assessment

Ability for self-caring activities were determined using the German version of the Barthel-Index (BI) [18]. The BI point’s (pts) range is 0–100 points, with 100 indicating independence in all activities of daily living. Montreal Cognitive Assessment (MoCA) [19] and Mini-Mental State Examination (MMSE) [20] were used to evaluate cognitive function in MHH and in MHB, respectively. Patients with total scores below 26 were considered as cognitively impaired. Depressive symptoms were assessed by the Depression in Old Age Scale (DIA-S; no depression 0–2 points, suspected depression 3 point, and probable depression 4–10 points) [21] in MHH and Geriatric Depression Scale-15 (GDS-15; 0–5 points: normal, 5–10 points: mild to moderate depression, and 11–15 points: severe depression) [22] in MHB.

### Measurement of C-reactive protein

CRP was analyzed according to standard procedures directly at hospital admission or the day after. Levels between 0.0 and 0.49 mg/dl indicate no inflammation (=normal values) whereas levels between 0.5 and 3.0 mg/dl are considered as mild inflammation and ≥3.0 mg/dl as moderate to severe inflammation [12, 13]. To compare the percentage of patients with food intake <50% and <75% of demands across the CRP levels, we used CRP groups as follows: 0.0–0.99 mg/dl, 1.0–1.99 mg/dl, 2.0–2.99 mg/dl, 3.0–4.99 mg/dl, 5.0–9.99 mg/dl and ≥10.0 mg/dl.

### Data analysis

The statistical analysis was completed with SPSS statistical software (SPSS Statistics for Windows, IBM Corp, Version 27.0, Armonk, NY, USA). Continuous variables are expressed by their means and standard deviations (SDs) for normally distributed variables and median values with interquartile ranges (IQR) for non-normally distributed data. Categorical variables are expressed as absolute numbers and relative frequencies (%). To explore the differences in CRP levels across the food intake levels and MNA-SF categories, we performed the Kruskal–Wallis test followed by pairwise comparison. Comparison of the proportions of patients with food intake <50% and <75% of requirements between CRP groups was performed by using the one-way ANOVA Tukey test or Chi-square test as appropriate. A *P* value <0.05 was accepted as the limit of significance.

## Results

### Subject characteristics

The baseline characteristics of the study population are displayed in Table 1. Of the total population, mean age of 82.2 ± 6.6 years (241 females), 27% had no inflammation and 40% had mild and 33% moderate to severe inflammation. According to MNA-SF, 23% of patients were malnourished and 53% at risk of malnutrition. Of the 377 patients, 82 (22%) had food intake <50% of requirements, in which, 37 (45%) demonstrated moderate to severe inflammation whereas 29 (35%) had mild and 16 (20%) no inflammation in this group.

**Table 1:** Characteristics of the study population.

	Total population (*n* = 377)
Gender	
Female (n; %)	241 (64)
Male (n; %)	136 (36)
Age (y)	82.2 ± 6.6
Height (m)	1.66 ± 0.08
Actual body weight (kg)	73.2 ± 17.5
BMI (kg/m^2^)	26.5 ± 5.8
MNA-SF, Median (IQR)	9 (8–11)
Malnourished (n; %)	84 (23)
At risk of malnutrition (n; %)	200 (53)
Normal nutritional status (n; %)	90 (24)
Barthel-Index on admission, Median (IQR)	45 (35–60)
MoCA, Median (IQR)	20 (16–23)
MMSE, Median (IQR)	26 (23–28)
DIA-S, Median (IQR)	3 (1–5)
GDS-15, Median (IQR)	0 (0–1)
Food intake	
>75% of needs (n; %)	144 (38)
50–75% of needs (n; %)	148 (40)
<50% of needs (n; %)	82 (22)
CRP (mg/dl)	3.8 ± 1.4
No inflammation (0.0–0.49 (mg/dl), n; %)	99 (27)
Mild inflammation (0.5–3.0 (mg/dl), n; %)	152 (40)
Moderate to severe inflammation (≥3.0 (mg/dl), n; %)	126 (33)

Values are given as mean ± SD, number (%) or median (IQR, interquartile range). Cognitive function and depression were evaluated using MoCA and DIA-S in 200 patients and using MMSE and GDS-15 in 177 patients.

*BMI* body mass index, *MNA-SF* Mini Nutritional Assessment Short, *MOCA* Montreal Cognitive Assessment, *MMSE* Mini-Mental State Examination, *DIA-S scores* Depression in Old Age Scale, *GDS-15* 15-item Geriatric Depression Scale, *CRP* C-reactive protein.

### Association of inflammation with food intake and nutritional status

**Fig. 1:**
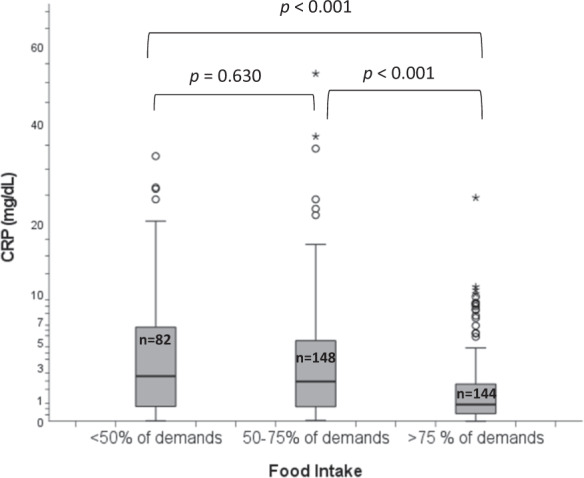
Comparison of CRP values across the food intake levels in total population (*n* = 377). CRP, C-reactive protein.

Significant differences in median CRP concentrations across food intake levels were observed (Fig. 1). Patients with food intake <50% of requirements had a significantly higher median CRP level (2.3 mg/dl) compared with the group of patients with food intake >75% of requirements (0.7 mg/dl, *P* < 0.001, Fig. 1a). However, there were no significant differences in median CRP levels across categories of MNA-SF (median CRP levels in malnourished patients: 1.4 (IQR: 0.4–4.3) mg/dl, at risk of malnutrition: 1.4 (IQR: 0.5–4.6) mg/dl and normal nutritional status: 1.1 (IQR: 0.4–4.1) mg/dl).

**Fig. 2:**
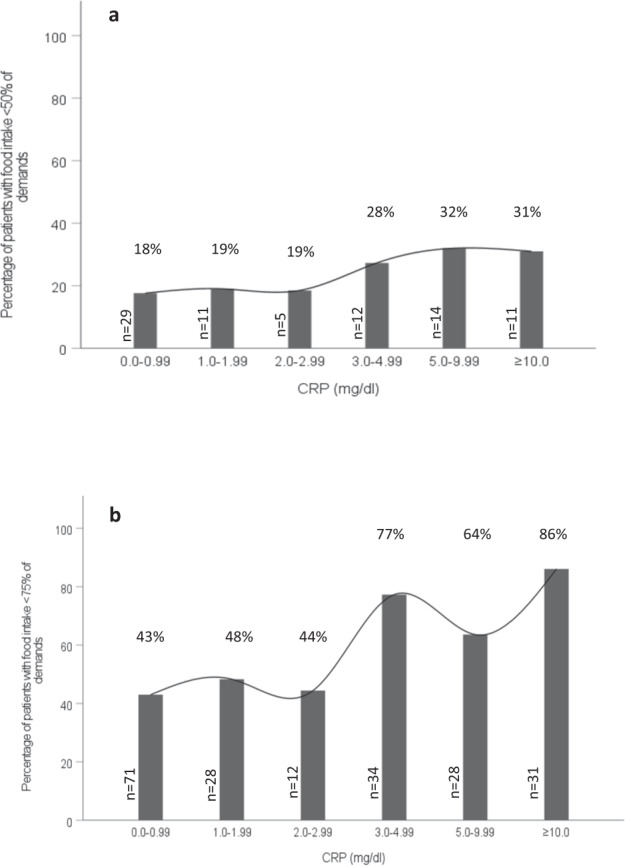
Percentage of patients with (a) food intake <50% (*n* = 82) and (b) food intake <75% of demands (*n* = 204) across the categories of CRP levels. CRP, C-reactive protein.

In addition, the proportion of patients with food intake <50% of demands were almost similar at a CRP level of 0.0–0.99 mg/dl, 1.0–1.99 mg/dl and 2.0–2.99 mg/dl, and became progressively higher with a CRP level of 3.0 mg/dl and above (from 19% at CRP levels 2.0–2.99 mg/dl to 28% at CRP levels 3.0–4.99 mg/dl; *P* = 0.062, Fig. 2a). There were significant differences in the proportion of patients with food intake <50% of requirements between CRP level <3 mg/dl and ≥3 mg/dl (18% vs. 30%, respectively; *P* = 0.017).

We also observed similar results using food intake <75% of requirements. The proportions of patients with food intake <75% of demands reached to 77% at CRP levels 3.0–4.99 mg/dl compared to 44% at CRP levels <3.0 mg/dl (*P* = 0.057, Fig. 2b). Further, significant differences in the proportion of patients with food intake <75% of requirements between CRP level <3 mg/dl and ≥3 mg/dl (45% vs. 75%, respectively; *P* < 0.001) were observed.

## Discussion

In the present study, inflammation as measured by serum-CRP, was associated with low food intake in older hospitalized patients. The group with serum-CRP levels above 3.0 mg/dl had a markedly higher proportion of patients with low food intake; i.e., <50% and <75% of the requirements. In the original cross-sectional study among older hospitalized patients (MHH), Sieske et al. already reported that almost 70% of patients with CRP > 3.0 mg/dl had food intake <75% of demands [5]. In addition, results of a randomized, double-blind nutritional intervention study among 455 hospitalized older patients indicated that older patients with elevated CRP levels (CRP > 1 mg/dl) had significantly lower energy intake [6]. It is noteworthy that authors of the aforementioned studies did not assess at what level of serum-CRP, older hospitalized patients are at increased risk of impaired food consumption. In the current study, a CRP-level of ≥3.0 mg/dl appears to be a reasonable threshold when food intake is impaired in older patients. Thus, this threshold may indicate the level of acute inflammation that could be used for the fulfillment of the inflammation etiologic criterion in the recently introduced GLIM diagnosis of malnutrition, especially for hospitalized older patients.

The original GLIM construct described inflammation as an etiologic criterion for malnutrition mainly related to prolonged catabolism and anorexia during acute disease or chronic diseases [8]. After the GLIM format was described, some authors have utilized relatively low degrees of inflammation, and independently from any diagnosis of chronic disease, for the fulfillment of the GLIM inflammation criterion. For example, in a recent cohort study of 534 community-dwelling older participants, malnutrition was diagnosed according to the GLIM criteria using an interleukin-6 cut-off value within the normal range of healthy persons (interleukin-6 > 3.84 pg/mL in men and >2.99 pg/mL in women) [23]. In another prospective cohort study of community-dwelling Chinese subjects (median age: 72 years), high-sensitivity CRP cut-off levels of ≥0.32 mg/dl in men and ≥0.38 mg/dl in women were used for the diagnosis of malnutrition according to GLIM [24]. Inflammation contributes to malnutrition through anorexia with reduced food intake, as well as through tissue catabolism leading to muscle and fat wasting. Not only the degree of inflammation, but also the duration of the inflammatory exposure needs to be considered. Modest chronic elevations may have long-term devastating effects on the nutritional status, comparable with the catabolic effects of shorter exposures of highly active inflammation.

The publication of the GLIM diagnosis of malnutrition is an important step toward a uniform and globally accepted definition. However, the un-clear definition of the inflammation criterion, including the lack of distinction between acute high-grade and chronic low-grade inflammation, is a weakness of the GLIM diagnostic framework. Applying dissimilar inflammation criteria may over- or under-estimate malnutrition. This current weakness is of prior relevance. In clinical practice, the criterion of inflammation is easier to obtain compared to the reduced food intake criterion. Clarity would likely further facilitate the use of the GLIM diagnostic format. The current pilot study may be a step in this direction. Consequently, more research is required to determine clinically relevant thresholds of acute and chronic inflammation with regard to acute and chronic malnutrition.

Some limitations of the study need to be addressed. First, it is the design as a post hoc analysis from previous studies with two different methods to determine low food intake. There is no gold standard and validated tool for evaluating food intake in older adults; however, we used plate diagram, which was shown to be valid in the clinical setting [25] and a single question about food intake proposed by NRS-2002 which is clinically relevant [16]. Second, we examined very old and acutely ill patients who suffered from multiple comorbidities including chronic disease. Therefore, it is not possible to separate the effect of acute and chronic inflammation in this population. Since the etiology of malnutrition in older persons is commonly multifactorial, it is very likely that there are other reasons than acute inflammation causing low food intake in our study population; therefore, residual, uncontrolled confounding cannot be excluded. Therefore, future prospective studies are needed in patients where disease-related inflammation is the major cause of malnutrition.

## Conclusion

A serum-CRP of 3.0 mg/dl appears to be a reasonable threshold of acute inflammation leading to reduced food intake to serve as an orientation with regard to the inflammation criterion of the GLIM diagnosis in acutely ill older patients.
